# Using electronic medication monitoring to guide differential management of tuberculosis patients at the community level in China

**DOI:** 10.1186/s12879-019-4521-2

**Published:** 2019-10-15

**Authors:** Ni Wang, Hui Zhang, Yang Zhou, Hui Jiang, Bing Dai, Miaomiao Sun, Ying Li, Amelia Kinter, Fei Huang

**Affiliations:** 1 National Center for Tuberculosis Control and Prevention, Chinese Center for Disease Control and Prevention, Beijing, 102206 China; 2 Jiangsu Provincial Center for Disease Control and Prevention, Nanjing, 210009 Jiangsu China; 3 Zhenjiang Center for Disease Prevention and Control, Zhenjiang, 212050 Jiangsu China; 4 PATH China Program, Beijing, 100600 China; 5 PATH HIV and Tuberculosis Program, 455 Massachusetts Ave, Suite 1000, Washington, DC, 20001 USA

**Keywords:** Tuberculosis, Electronic medication monitor, Patient management, China

## Abstract

**Background:**

In settings such as China, where universal implementation of directly observed therapy (DOT) is not feasible, innovative approaches are needed to support patient adherence to TB treatment. The electronic medication monitor (EMM) is one of the digital technologies recommended by the World Health Organization (WHO), but evidence from implementation studies remains sparse. In this study, we evaluated acceptance of the EMM among health care workers and patients while implementing the device for differential TB patient management at the community level.

**Methods:**

Zhenjiang City in Jiangsu Province was purposively selected for the study. All participating patients were allowed to select their preferred management approach. If patients declined to use the EMM, DOT was offered. The EMM was designed to hold 1 month of anti-TB drugs for once-daily dosing of fixed-dose combination (FDC) tablets. Patient EMM records were monitored monthly by a physician; if 20 to 50% of doses were missed twice, or more than 50% of doses were missed once, the patient was switched to DOT. The four physicians and five nurses involved in the study at four designated hospitals were surveyed using a structured questionnaire to assess their acceptance of the EMM.

**Results:**

From October 2017 through January 2018, 316 pulmonary TB patients were notified in the TB information management system, and 231 (73.1%) met the study enrollment criteria. Although 186 patients (80.5%) initially consented to use the EMM, 17 later refused to use it. Among the 169 patients who used the EMM, 15 (8.9%) were switched to DOT due to poor adherence, and the other 154 completed the treatment course. The median adherence rate was 99.3%. Surveyed health care workers from designated hospitals found the EMM acceptable, although eight of nine felt use of the device moderately increased their workload. However, the EMM program significantly reduced the workload of community physicians by reducing patient visits by 87.9%.

**Conclusions:**

This study demonstrated the acceptability of using an indigenously developed EMM for differential management of TB patients at the community level. However, more operational research should be conducted before introducing and scaling the technology throughout China.

## Background

According to the World Health Organization’s (WHO’s) 2018 Global Tuberculosis Report, China has the world’s second highest tuberculosis (TB) burden, with an estimated 889,000 cases (new and existing) in 2017, accounting for 8.9% of the global total [1]. Although the use of directly observed therapy (DOT) by health care workers is the most widely known approach to ensure treatment adherence, recent evidence indicates that the program is not being implemented systematically, resulting in lower patient adherence rates [2–4]. Poor adherence is cited as the primary reason for suboptimal clinical benefits of TB treatment, and it leads to worse clinical outcomes, including development of drug resistance, increased duration of infectivity, and transmission to others [5]. A systematic review in China found that only 50% of TB patients received DOT (20% from a health care worker and 30% from a family member), and 50% self-administered treatment (SAT) without any adherence support [6]. Meanwhile, evidence showed that SAT patients had poor adherence compared to those on DOT [7]. According to the 2010 national TB prevalence survey in China, 20% of TB patients treated in the public health system were lost to follow-up or were not taking their medication regularly [8].

The use of digital health technologies may help to reach the new global End TB targets [9]. According to the updated WHO treatment guidelines for drug-susceptible TB, three digital technologies are recommended: short message service (SMS, or text messaging), the electronic medication monitor (EMM), and video-observed treatment (VOT) [10]. The three digital technologies have individual strengths and weaknesses that determine their suitability for distinct circumstances. The EMM, which can operate without mobile broadband Internet coverage, is currently the most accessible, affordable, and scalable solution in resource-limited settings [11]. Hence, between 2009 and 2015, the Chinese Center for Disease Control and Prevention (China CDC) explored the use of an indigenously developed EMM for improving treatment adherence. Under trial conditions, EMM use decreased the frequency of missed doses by 40 to 50% compared to SAT [7]. A usability study conducted in rural China with the EMM also showed a high level of user performance, acceptability, and satisfaction among both TB patients and health care workers [12].To strengthen community-based case management, China’s National 13th Five Year Plan on TB Control (2016–2020) included adoption of the EMM as part of TB case management approaches [13].

Feasibility of EMM implementation, user acceptability, and qualitative assessments at scale are important considerations, but evidence from implementation studies remains sparse [14, 15]. To extend the use of the EMM, the China CDC developed an EMM scale-up plan for three provinces involving 138 counties. Since this is the first large-scale use of the EMM in China, on behalf of the China CDC, we conducted this operational research to evaluate the acceptance of the EMM among health care workers and patients while implementing the device in actual routine practice by the local TB program.

## Methods

### Study design

This is a cohort study using quantitative data collection methodologies to evaluate the acceptability of the EMM among health care workers and patients. A self-administered questionnaire was used to assess health care workers’ acceptance of the device in relation to its operation, usefulness, and effect on their workload. Acceptability among patients was assessed based on the proportion of eligible patients who chose to use EMM and the factors associated with this choice, as well as the proportion who switched to DOT and the associated factors.

Zhenjiang City in Jiangsu Province was purposively selected for the study. The city has well-established TB control and prevention services, with sufficient human resource capacity. In addition, in the 3 years prior to the study, approximately 1200 active pulmonary TB cases were detected annually and notified in the TB information management system (TBIMS), allowing the study to be implemented quickly to provide results prior to scale-up. Zhenjiang is located in eastern China, has four designated TB hospitals, and has a total population of about 3 million [16].

Each active TB case was tested using Cepheid’s GeneXpert® system to identify drug resistance, and all the patients were treated with standard short-course chemotherapy for 6 to 8 months according to China’s national TB control program guidelines, using once-daily fixed-dose combination (FDC) tablets produced by a single manufacturer.

All the health care workers (four physicians and five nurses) from designated hospitals who were responsible for EMM use were surveyed through the use of self-administered questionnaires. Respondents sent the questionnaires anonymously to a designated email address, and the research group promised to not give the results to their direct superiors. In addition, the research group looked for differences in the total number of patient visits by community physicians depending on whether patients were using DOT or the EMM.

### Patient enrollment and withdrawal

Study participants who met the following criteria were enrolled in the study: active pulmonary TB patient notified between October 1, 2017, and January 31, 2018; receiving daily outpatient treatment; and having no communication impairment (mental, visual, auditory, or speech). The study excluded rifampicin-resistant patients and patients not being treated locally.

All eligible patients were advised to use the EMM by physicians at the designated TB hospitals but verbal consent was required. If patients consent to use EMM, they were required to sign a consent form later by the community physicians at the first home visit. If patients refused to use the EMM, the physicians would record the reasons. If patients stopped using the EMM midway through the study, the physicians would also record the reasons.

### Interventions

The EMM was designed to monitor treatment adherence throughout a one-month FDC regimen. The device components include a plastic box and an electronic module. The electronic module can be reused for at least three patient cycles (each patient being treated for up to 6 or 8 months), and use by each patient costs approximately US$5.

Nurses programmed the study devices to provide a medication reminder at the same time each day for each TB patient, at a time selected by the patient. The EMM recorded each time the patient opened the device, indicating the patient had taken his or her medication, and clinicians accessed the data from the EMM management system while the device was connected to a computer. TB patients were required to visit a designated hospital monthly for follow-up examination, review of their EMM data and treatment adherence by a physician, and collection of the next month’s anti-TB drugs. Based on the previous month’s medication adherence data, if < 20% doses were missed, the patient was counselled. If 20 to 49% of doses were missed, the frequency of home visits by village doctors was increased (once every 7 days for the rest of the treatment). And if 20 to 49% of doses were missed twice, or ≥ 50% of doses were missed once, then the patient was transitioned to DOT for the remainder of treatment [7].

Community physicians were required to visit patients enrolled in the EMM study once every 10 days during the intensive phase (the first 2 months of treatment) and once a month in the continuation phase to check on their health status and monitor EMM usage. A reading on a small LED screen in the EMM displayed the regularity with which the patient took his or her doses. In addition, the EMM data were regularly uploaded to the online EMM information management system (EMMIMS) by county-designated hospital staff so health care workers at all levels could query the medication adherence of TB patients within their jurisdiction.

### Data management and analysis

Case data were collected from all TB patients notified in the electronic TBIMS from October 1, 2017, to January 31, 2018. Data were collected on variables such as TBIMS code for basic management unit (BMU) (the hospitals which are established at the county level in China, in contrast to TB management units, which are established at the provincial, prefecture and county levels), registration number, sex, age, occupation, migrant status, treatment type (new or retreated), bacteriological results (bacteriologically confirmed or clinically diagnosed), and the treatment initiation date. Patients’ TBIMS data were matched to the EMMIMS, where uploaded case data were also collected from October 1, 2017, to January 31, 2018, including variables such as TBIMS code for BMU, registration number, sex, age, date of EMM initiation, date of EMM completion, missed doses, and days the patient should have taken medication. The two systems could be matched by the TBIMS code for BMU and registration number. Other variables, including reasons for declining or discontinuing use of the EMM, were collected from medical records by the research team.

A database was constructed and cleaned with Microsoft Excel, and analysis was conducted using SPSS version 19. The proportions of patients who were eligible for EMM use, who used the EMM, and who stopped using the EMM midway through the study were calculated. And among patients who used the EMM and who completed treatment, the average percentage of doses taken was calculated. Unadjusted and adjusted odds ratios (0.95 CI) were calculated using log binomial regression to summarize (infer) factors associated with a patient’s refusal to use the EMM at enrollment. Unadjusted and adjusted odds ratio (0.95 CI) were also calculated using log binomial regression to summarize (infer) factors associated with patients who were switched to DOT due to poor adherence. Age, sex, occupation, migrant status, treatment category, and diagnosis classification were considered in the model regardless of the unadjusted *p* value.

The workload of community physicians was evaluated by assessing the number of patient visits. In the DOT management program, the community physicians are required to visit a new TB patient 180 times (daily visits for 6 months) and a retreated TB patient 240 times (daily visits for 8 months). However, under the EMM management program, the physician visits are reduced to 10 visits for a new TB patient and 12 visits for a retreated TB patient (once every 10 days during the intensive phase followed by once per month in the continuation phase). If a patient is switched to the DOT program, the physician’s visits must revert back to the daily visits upon ceasing use of the EMM.

### Quality control

All the physicians and nurses received a detailed implementation standard operating procedure and were trained by the research team. Onsite technical support was conducted quarterly, and questions were addressed through social media networking platforms (WeChat) within 24 h.

## Results

### Patient characteristics

**Fig. 1:**
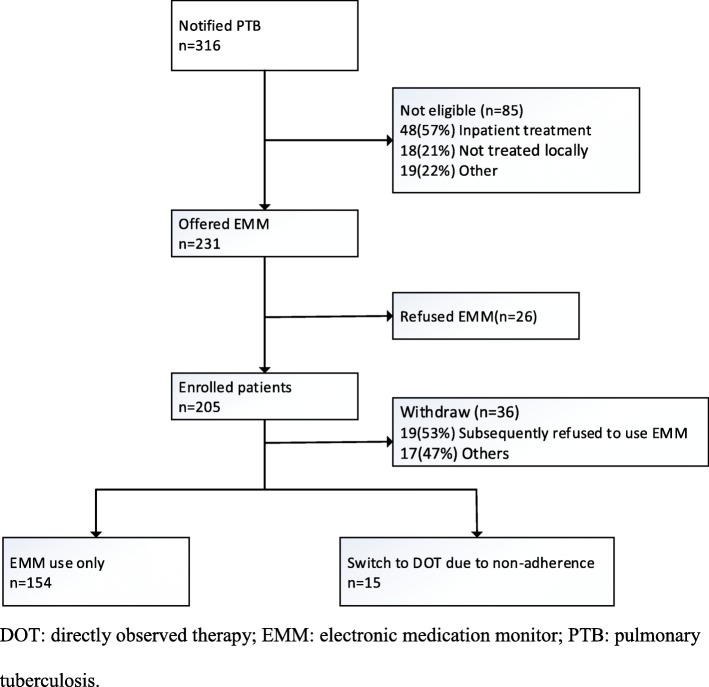
Flow chart depicting study enrollment and the outcome of using the EMM in the study city

From October 1, 2017, to January 31, 2018, 316 pulmonary TB patients were notified in TBIMS. Of these, 85 cases were excluded: 48 who required inpatient treatment, 18 who were not receiving local treatment, 7 who were identified with a communication impairment, 7 who received a non-TB diagnosis from their physician, 3 who died prior to treatment enrollment, and 2 who were lost to follow-up before treatment initiation. Eventually, 231 patients met the study’s enrollment criteria (Fig. 1).

Among the 231 study patients, the mean (SD) age was 56.7 (18.9) years, the minimum age was 12 years, and the maximum age was 89 years. In addition, 164 patients (71.0%) were males, 142 (61.5%) were farmers or migrant workers, 160 (69.3%) were local residents, 206 (89.2%) were new TB patients, and 115 (49.8%) were bacteriologically confirmed TB patients (Table 1).

**Table 1:** Factors associated with refusing to use the EMM among eligible TB patients in the study city (*n* = 231)

Factors	Total		Refused to use EMM		OR	(95% CI)	aOR^f^	(95% CI)
	n	(%)^a^	n	(%)^b^				
Sex								
Male	164	71.0	18	11.0	ref		ref	
Female	67	29.0	8	11.9	1.1	0.5–2.7	1.3	0.5–3.3
Age^c^								
< 44	54	23.4	4	7.4	ref		ref	
45–64	82	35.5	8	9.8	1.6	0.6–4.0	1.5	0.6–3.9
> =65	95	41.1	14	14.7	2.2	0.7–6.9	1.7	0.5–6.1
Occupation								
Farmer/migrant worker	142	61.5	17	12.0	1.2	0.5–2.8	1.2	0.5–3.0
Other	89	38.5	9	10.1	ref		ref	
Migrant^d^								
No	160	69.3	18	11.3	ref		ref	
Yes	71	30.7	8	11.3	1.0	0.4–2.4	1.6	0.5–2.9
Category								
New	206	89.2	22	10.7	ref		ref	
Retreated	25	10.8	4	16.0	1.6	0.5–5.1	1.0	0.3–3.5
Classification								
Bacteriologically confirmed	115	49.8	20	17.4	3.9^e^	1.5–10.0	3.7^e^	1.4–9.8
Clinically diagnosed	116	50.2	6	5.2	ref		ref	

*TB* Tuberculosis, *EMM* Electronic medication monitor, *OR* Odds ratio, *aOR* Adjusted odds ratio, *CI* Confidence interval

^a^Column percentages

^b^Row percentages

^c^Only one patient was under 15 years old, so < 15 group was merged into < 44 group

^d^Migrant defined as patient coming from another county

^e^Statistically significant

^f^Even though some variables didn’t show statistical significance in univariate analysis, considering the important influence of patient’s background and diagnosis in the treatment management, we included all the variables in the multivariable analysis

### Acceptability of the electronic medication monitor among study patients

Of the 231 patients enrolled in the study, 26 patients (11.3%) refused to use the EMM initially. Another 19 patients (8.2%) subsequently refused to use the EMM during their treatment, and their median number of days of using the EMM was 109 (minimum 6 days, maximum 174 days). A total of 186 patients (80.5%) consented to use the EMM. Among these patients, 17 (9.1%) were later withdrawn because of inpatient treatment needs, death, departure from the city, or loss to follow-up. Eventually, 169 patients (90.8%) remained in the cohort (Fig. 1).

Patients who were diagnosed as bacteriologically confirmed TB were much more likely to refuse to use the EMM [aOR: 3.7 (95% CI: 1.4–9.8)] (Table 1).

### Results of differential patient management

Among the 169 patients in the cohort using the EMM, 15 (8.9%) switched to DOT due to poor adherence with treatment as demonstrated by the EMM; their median number of days of using the EMM was 136 (minimum 43 days, maximum 160 days). Among the 154 patients who used the EMM throughout the treatment course, the median adherence rate (average percentage of doses taken) was 99.3% (minimum 83.4%, maximum 100.0%). There was 100% adherence among 61 patients (39.6%), 90–100% adherence among 91 patients (59.1%), and 80–90% adherence among 2 patients (1.3%).

Compared to farmers or migrant workers, people in other occupations were more likely to switch to DOT due to poor adherence [aOR: 4.2 (95% CI: 1.1–15.4)]. Also, migrants more often demonstrated poor adherence and switched to DOT than did patients who came from the local county [aOR: 8.4 (95%CI: 2.3–30.6)]. And retreated TB patients were more likely than new TB patients to switch to DOT due to poor adherence [aOR: 7.6 (95% CI: 1.1–51.0)] (Table 2).

**Table 2:** Factors associated with TB patients who were switched to DOT due to non-adherence in the study city (*n* = 169)

Factors	Total		Switched to DOT		OR	(95% CI)	aOR^f^	(95% CI)
	n	(%)^a^	n	(%)^b^				
Sex								
Male	123	72.8	9	7.3	ref		ref	
Female	46	27.2	6	13.0	1.9	0.6–5.7	1.9	0.5–7.0
Age^c^								
< 44	44	26.0	7	15.9	2.6	0.7–9.5	1.9	0.4–9.7
45–64	66	39.1	4	6.1	0.9	0.2–3.7	0.9	0.2–4.3
> =65	59	34.9	4	6.8	ref		ref	
Occupation								
Farmer/Migrant worker	100	59.2	4	4.0	ref		ref	
Other	69	40.8	11	15.9	4.6^e^	1.4–15.0	4.2^e^	1.1–15.4
Migrant^d^								
No	119	70.4	4	3.4	ref		ref	
Yes	50	29.6	11	22.0	8.1^e^	2.4–26.9	8.4^e^	2.3–30.6
Category								
New	151	89.3	12	7.9	ref		ref	
Retreated	18	10.7	3	16.7	2.3	0.6–9.1	7.6^e^	1.1–51.0
Classification								
Bacteriologically confirmed	75	55.6	6	8.0	ref		ref	
Clinically diagnosed	94	44.4	9	9.6	1.2	0.4–3.6	1.4	0.4–5.3

*TB* Tuberculosis, *DOT* Directly observed therapy, *OR* Odds ratio, *aOR* Adjusted odds ratio, *CI* Confidence interval

^a^Column percentages

^b^Row percentages

^c^Only one patient was under 15 years old, so < 15 group was merged into < 44 group

^d^Migrant defined as patient coming from another county

^e^Statistically significant

^f^Even though some variables didn’t show statistical significance in univariate analysis, considering the important influence of patient’s background and diagnosis in the treatment management, we included all the variables in the multivariable analysis

### Acceptability of the electronic medication monitor among health care workers

All nine surveyed health care workers (four physicians and five nurses) agreed the EMM was useful to both patients and clinicians. In addition, the nurses, who programmed each EMM to meet individual patient needs, considered the operation of the EMM to be acceptable. Eight health care workers felt their workload increased while using the EMM, but seven of the eight felt the increase was only moderate (Table 3).

**Table 3:** Results from the structured questionnaire survey with participating physicians and nurses in the study city

Question	Number of nurses who agreed (*n* = 5)	Number of physicians who agreed (*n* = 4)	Total
EMM was useful to patients	5	4	9
EMM was useful to physicians	5	4	9
Operation was acceptable	5	N/A	5
Workload increased while using the EMM	4	4	8
Increased workload was moderate	3	4	7

*EMM* Electronic medication monitor, *N/A* Not applicable

The median time to set up the EMM for each patient at the first visit, including time for training and programming the medication reminder time, was 15 min (minimum of 5 min, maximum of 20 min). Patient follow-up visits took an average of 7 min (range of 3 to 10 min), including time for providing counseling based on the collected data and setting the EMM for the next visit.

If the 205 study patients were enrolled in the DOT program, community physicians would have had to make a total of 38,160 visits. But with the EMM management program, the number of patient visits declined dramatically to 4604, a decrease of 87.9% (Table 4).

**Table 4:** Differences in the workload of community physicians when using DOT management or EMM management in the study city (*n* = 205)

Category	DOT management		EMM management					Reduction (%)^a^
	n	Visits	EMM use only		Switched to DOT midway		Total visits	
			n	Visits	n	Visits		
New	184	33,120	155	1550	29	2205	3755	88.7
Retreated	21	5040	16	192	5	657	849	83.2
Total	205	38,160	171	1742	34	2862	4604	87.9

*DOT* Directly observed therapy, *EMM* Electronic medication monitor

^a^Reduction between DOT management visits and EMM management total visits

## Discussion

This study found high patient acceptability with the EMM; among the eligible patients, only 11.3% of patients (26/231) refused to use the device initially, and another 8.2% (19/231) subsequently refused to use the device. The total acceptance rate was 80.5% (186/231). Positive bacteriological patients were more likely to refuse to use the device in this study, and further research is required to identify the reasons for this correlation. Moreover, similar to other studies [4, 17, 18], our study noted that retreated TB patients were more likely to have poor adherence compared to new TB patients, migrant TB patients were more likely to have poor adherence compared to patients from the local county, and farmers or migrant workers were more likely to have stronger treatment adherence results compared to those in other occupations, but since there were only 15 patients who switched to DOT due to poor adherence, the results need to be confirmed by further studies. Another important finding was that most patients who stopped using the EMM did so in the last 2 months of treatment; a systematic review likewise found that the effectiveness of interventions to improve adherence decreased by 1.1% each month [19], suggesting that patients need more interventions to enhance medication adherence in the middle and late stages of treatment.

Additionally, results from data collection with the structured questionnaire suggested good acceptance of the EMM among health care workers. Because the EMM did not provide real-time data transmission, which limited physicians’ timely access to patient dosing data, the physicians were unable to offer timely intervention to correct dosing behaviors. However, the LED screen has been associated with improved adherence [20], and EMMIMS, the Internet-based system for uploading data, allowed users at different levels to query and review patient medication adherence data.

The EMM in this study not only provided patient medication reminders but also generated dosing data that helped health care providers identify patients who were non-adherent to therapy. We successfully implemented the differential patient management program, as only 8.9% of patients (15/169) were found non-adherent by EMM and shifted to DOT. Among the other 91.1% of patients (154/169), who used the EMM and completed treatment, the median adherence rate was 99.3%. Moreover, EMM use significantly reduced the workload of community physicians compared to DOT. The resources saved by not giving DOT to the adherent patients could help to strengthen management of non-adherent patients—for example, by implementing DOT, tracking the interrupted patients, increasing counselling, and providing more home visits. Several studies [7, 14, 19, 21, 22] have demonstrated the effectiveness of an EMM in improving medication adherence among TB patients. Furthermore, two studies [23, 24] have suggested that implementing differential patient management programs through the use of an EMM, using a package of interventions tailored to each patient’s needs and values, is more likely to improve TB outcomes. While the most appropriate thresholds for switching to DOT need to be discussed further, according to a recent study, less than 90% medication adherence among TB patients is a significant risk factor for unfavorable outcomes [25]. If we had followed this criterion, 10.1% of patients (17/169) in this study would have been switched to DOT, an increase of 1.3 percentage points.

Development and implementation of digital health products at a large scale requires continued engagement of both TB patients and health care workers, in addition to innovators, funders, policymakers, advocacy groups, and affected communities [9]. Although use of the EMM greatly reduced the workload of community physicians, county clinics expended additional resources on training, EMM configuration, data downloading and uploading, and data-driven counseling. Even though most health care workers reported that use of the EMM only moderately increased their workload, it is important to note that the patient management workload was partially transferred from the community to the county, and it is important to think about optimizing resource allocations accordingly. Another study from China found that physicians from county designated hospitals largely ignored the adherence data when deciding whether to switch patients to a more intensive case management approach because of insufficient financial incentives to do so [12]. Moreover, it was noted that health care workers spent nearly twice as much time with patients during their first visit than during follow-up visits, suggesting that health care workers could benefit from training materials or videos on the EMM.

There were several limitations of this study. First, the intervention was implemented in only one prefecture, located in eastern China, where the level of economic development is higher than the national average and the health care community generally has sufficient human resources. Therefore, the results obtained cannot predict the success of national scale-up but support efforts to further study and expand use of the EMM. Second, the study lacked cost-effectiveness and intervention impact analyses, which would yield essential information for considering large-scale use of the EMM [26]. A cluster-randomized, controlled trial currently being undertaken in China by the China CDC will assess whether an EMM-based treatment strategy can improve clinical outcomes for TB patients [27]. Third, the study did not collect data from patients about their perceptions of EMM use. Based on this study, the authors recommend more operational research, including studies on coverage of EMM use, adherence using the EMM, and TB treatment outcomes under programmatic conditions in more areas. Additional research should also assess patients’ perceptions of EMM use and include cost minimization analysis as well as cost-effectiveness analysis, to provide public health planners in low-resource settings with adequate data to evaluate the return on investment of treatment adherence technology for broader implementation [28].

Despite these limitations, the authors believe this is the first operational research to assess the acceptability of an EMM that does not provide real-time data transmission in actual routine practice by the local TB program. Based on the findings from this research, the same EMM device has been used in three selected provinces since Jun 2018, and since January 2019, all 138 counties from the three provinces have started using the EMM.

## Conclusions

In settings such as China, where universal use of DOT is not feasible, innovative approaches are needed to support patient adherence to TB treatment. Our study successfully used an EMM to guide differential patient management at the community level in China, and the results support further expansion of this locally developed, low-cost, quality-assured device under the national TB control program. Evidence from implementation studies is sparse, however. To advance this intervention, more operational research needs to be conducted on the coverage of EMM, adherence using the EMM under programmatic conditions in more areas, cost-effectiveness, and the impact of the intervention. The results are needed to consider introduction and scale-up of the technology countrywide.

## Data Availability

The datasets used and/or analyzed during the study will be shared upon request. Please contact to Dr. Fei Huang (email: huangfei@chinacdc.cn).

## Abbreviations

aOR: Adjusted odds ratio
CI: Confidence interval
DOT: Directly observed therapy
EMM: Electronic medication monitor
EMMMIS: EMM information management system
FDC: Fixed-dose combination
OR: Odds ratio
SMS: Short message service
TB: Tuberculosis
TBIMS: TB information management system
VOT: Video observed treatment
WHO: World Health Organization
